# Melatonin supplementation and outcomes of assisted reproductive technology: a systematic review and meta-analysis

**DOI:** 10.1186/s12884-025-08503-1

**Published:** 2025-11-25

**Authors:** Hongying Tang, Jie Hao, Bin Xu, Yonggang Wang, Yanping Li, Jing Zhao

**Affiliations:** Reproductive Medicine Center, Xiangya Hospital, Central South University, 87 Xiangya Road, Changsha, Hunan Province P.R. China

**Keywords:** Melatonin, Assisted reproductive technology (ART), *In vitro* fertilization (IVF), Clinical pregnancy

## Abstract

**Background:**

Melatonin (MT) is involved in the regulation of various important biological processes related to reproduction. Many studies have investigated the effects of MT supplementation on outcomes following assisted reproductive technology (ART), yielding conflicting results. The aim of this systematic review was to synthesize evidence from clinical studies regarding the impact of MT on the key outcomes of ART.

**Methods:**

PubMed, Embase, Web of Science, and Google scholar were searched. Clinical trials that studied the effect of MT supplementation on outcomes following ART and published in English from inception to April 2020, were included. One author assessed the risk of bias in the studies using the Cochrane Collaboration checklist. Dichotomous outcomes were analyzed as risk ratios (RR) using the Mantel-Haenszel statistical method and a random/fixed effect model. Continuous outcomes were analyzed as Mean Difference (MD) using the Inverse Variance statistical method. The funnel plot was used to assess the publication bias.

**Results:**

Eleven studies conducted between 2008 and 2019 were included in this meta-analysis. Clinical pregnancy rate (CPR), live birth rate (LBR), Miscarriage rate (MR), fertilization rate (FR), Number of oocytes retrieved, MII oocytes, top-quality embryos was reported in 10, 3, 6, 7, 9, 8, and 6 studies, respectively. MT supplementation significantly increased the CPR (RR, 1.24; 95% confidence interval [CI], 1.04, 1.47), the number of MII oocytes (MD, 1.39; 95% CI, 0.74, 2.04), the number of top-quality embryos (MD, 0.56; 95% CI, 0.24, 0.88), and the FR (4 studies with RR, 1.10; 95% CI, 1.03, 1.17; 3 studies with MD, 0.13; 95% CI, 0.01, 0.24). However, there was no significant difference in LBR (RR, 1.23; 95% CI, 0.85, 1.80), the number of oocytes retrieved (MD, 0.58; 95% CI, -0.12, 1.27), and the MR (RR, 0.96; 95% CI, 0.50, 1.82). When studies were sub-grouped by the control interventions, either myoinositol(MI) plus folic acid (FA) or placebo/no treatment, MT supplementation increased number of MII oocytes (MT + MI + FA vs. MI + FA, MD, 0.91; 95% CI, 0.40, 1.41; MT vs. Placebo/no treatment, MD, 2.06; 95% CI, 0.73, 3.39) and number of top-quality embryos (MT + MI + FA vs. MI + FA, MD, 0.70; 95% CI, 0.24, 1.16; MT vs. Placebo/no treatment, MD, 0.33; 95% CI, 0.11, 0.54), while the CPR remained comparable between groups(MT + MI + FA vs. MI + FA, RR, 1.22; 95% CI, 0.96, 1.54; MT vs. Placebo/No treatment, RR, 1.26; 95% CI, 0.97, 1.62). When sub-group analysis was performed basing on women’s characteristics, MT supplementation showed no significant beneficial effect on CPR in women with polycystic ovary syndrome (PCOS) (RR, 1.18; 95% CI, 0.92, 1.52), normal ovarian function (RR, 1.15; 95% CI, 0.87, 1.53), or a history of low fertilization rates or poor-quality embryos (RR, 1.71; 95% CI, 0.95, 3.07). However, MT supplementation increased the number of MII oocytes in women with PCOS (MD, 0.97; 95% CI, 0.22, 1.73), but this benefit was not observed in women with normal ovarian function (MD, 1.49; 95% CI, -0.33, 3.31).

**Conclusions:**

As the outcomes of ART were influenced by multiple factors, MT supplementation may not significantly improve clinical pregnancy or live birth rate. However, MT appears to have a positive effect on oocyte and embryo quality, particularly in women with PCOS or decreased ovarian reserve (DOR), at least to some extent. Nevertheless, further well-designed, large-scale studies are needed before MT can be recommended for routine use in clinical practice.

**Supplementary Information:**

The online version contains supplementary material available at 10.1186/s12884-025-08503-1.

## Introduction

Since the birth of the first test-tube baby in 1978, assisted reproductive technology (ART) has made significant progress and is now widely used as an effective treatment for infertility [1]. However, current reports indicate that the clinical pregnancy rate (CPR) and live birth rate (LBR) remain suboptimal, averaging approximately 50% and 30%, respectively. The cumulative live birthrates (CLBR) across 3 ART cycles is around 51% [2]. Consequently, improving the success rate of ART remains the central focus of research in the field.

In the process of ART, failures in fertilization, embryo development, and implantation are often attributed to poor oocyte quality [3]. Although the underlying mechanism leading to poor oocyte quality are not yet fully understood, oxidative stress has been implicated as a contributing factor, potentially compromising female reproductive capacity [4]. Reducing oxidative stress in oocyte caused by reactive oxygen species (ROS) has been associated with improved embryo quality. Therefore, the use of anti-oxidants is proposed to mitigate the detrimental effects of excessive ROS and improve the success rate of ART.

Melatonin (MT)is a hormone primarily secreted by the pineal gland. MT and its metabolites can directly neutralize ROS in human tissues, upregulate the gene expression of various antioxidant enzymes, and reduce oxidative damage to cells [5].

Therefore, many clinics have incorporated MT supplementation into routine practice, and have investigated its potential effects on reproductive outcomes. Several studies have reported improvements in oocyte quality, embryo development, and ART outcomes [6–8]. In contrast, other studies have failed to demonstrate any benefit of MT supplementation in infertile women undergoing ART [9]. So far, two systematic reviews have examined the effects of MT supplementation on reproductive outcomes, yielding inconsistent results. One meta-analysis, which included five studies, failed to show significant benefits of MT supplementation on key reproductive outcomes [10]. The other study has concluded that supplementation of MT may significantly improve the CPR [11].

Therefore, it was necessary to evaluate whether MT supplementation has a beneficial effect on ART outcomes. This systematic review and meta-analysis aim to clarify the impact of MT supplementation on key reproductive outcomes in women undergoing ART.

## Methods

This systematic review and meta-analysis was conducted in accordance with the PRISMA guidelines. There was no completing interest.

Search strategy.

A comprehensive literature search was conducted in PubMed, Embase, Google Scholar and the Cochrane Library from inception to April 2020. The search strategy included three main categories of keywords: terms related to assisted reproductive technologies (e.g. assisted reproductive technology/ART/in vitro fertilization/IVF/intracytoplasmic sperm injection/ICSI); terms related to melatonin (e.g. melatonin/melatonin supplementation/MT); and terms related to outcomes of IVF/ICSI (live birth, clinical pregnancy, fertilization). The keyword subsets were combined with “AND” to refine the search and retrieve more literatures. Studies published in non-English were excluded.

### Study selection and data extraction

Two reviewers assessed the eligibility of the identified studies independently, and a third reviewer was consulted when there is discrepancy. Studies evaluating MT supplementation in women undergoing ART were included and analyzed. The primary outcomes of interest were LBR, CPR, number of oocyte/MII oocyte, number of top-quality embryos, fertilization rate (FR) and miscarriage rate (MR).

Data from each eligible study were extracted and recorded in a 2 × 2 table. For studies with incomplete data, we contacted the corresponding author or calculated the missing data from the available information when possible. In addition, information on study design, interventions, sample size, inclusion and exclusion criteria, usage of MT, and all reported outcomes was systematically recorded.

### Data analysis

RevMan 5.3 (Cochrane Collaboration, Oxford, UK) was applied for statistical analysis. The Risk Ratios (RRs) were calculated for binary outcomes using 2 × 2 tables, while mean differences (MDs) were used for continuous outcomes. Forest plots were used to assess the heterogeneity among the included studies graphically and the *l*^*2*^ statistic was used to quantitatively evaluate the degree of heterogeneity. The precision of the effect estimates was assessed using 95% confidence interval (CI), calculated through either fixed-effect or random-effect model. The methodological quality of the included studies was evaluated using the Newcastle-Ottawa Quality Assessment Scale. Due to the low statistical power of Chi-squared(X^2^) test for heterogeneity in meta-analysis, particularly in studies with small sample size, *a P* value of < 0.10 was considered as statistically significance.

## Results

### Studies selection and characteristics

The literature search yielded 160 citations. After screening titles and abstracts, 103 irrelevant studies were excluded. Of the remaining 57 articles, 44 studies were excluded because they did not report outcomes of IVF/ICSI or were not relevant to our subject. Additionally, two studies were system review and meta-analysis and were therefore excluded. The study selection flow diagram is shown in Supplemental Table 1.

The characteristics of the included studies are summarized in Table 1. A total of 11 prospective studies (1241 participants) were included in this meta-analysis. Among them, 10 studies reported clinical pregnancy rate (593 participants in intervention group and 516 in control group); 3 studies reported live birth rate (190 vs. 101 participants); 6 studies reported miscarriage rate (139 vs. 102 participants); 7 studies reported fertilization rate (172 vs. 176 participants), 9 and 8 studies reported the number of oocyte retrieved and MII oocyte(545 vs. 465 participants, 425 vs. 425 participants), and 6 studies reported the number of top-quality embryo (330 vs. 259 participants). In 7 studies, the control group received placebo or no treatment (269 participants). In 3 studies, standard treatment with myoinositol plus folic acid (MI + FA) was used (247 participants). One study did not report the control group intervention.

**Table 1:** The characteristic of included studies

Study	Country	Type of study	Treatment	Sample size	Inclusion criteria	Exclusion criteria	Usage of melatonin	Control	Outcome
Tamura et al. 2008	Japan	Prospective	GnRH-a IVF-ET Day 2 ET	115	FR < 50% in the previous IVF; nonsmoker; free from major medical illness	Myoma; adenomyosis, a congenital uterine anomaly, ovarian tumors, use of any medication, inclusing steroidal or nonsteroidal.	3 mg MT daily from the 5th day of the previous cycle until the day of oocyte retrieval	None	FR; CPR
Rizzo et al. 2010 [9]	Italy	Prospective	GnRH-a IVF-ET ET time/number NR	65	Age 35–42; low oocyte quality detected in the previous IVF cycle	NR	2 g MI twice with 200 mg FA and 3 mg MT from the day of GnRH-a treatment	2 g MI twice with 200 mg FA	CPR, MR, FR, No. of oocyte, MII, top quality embryo
Eryilmaz et al. 2011[18]	Turkey	Prospective RCT	GnRH-a IVF-ET Day 3, ET number 1–3	60	Disturbed sleep status; UI; without ovulatory and hysterosalpingography or laparoscopy problem	Chronic drug usage, history of IVF failure, hypertension, DM, uterine myoma, ovarian cyst, and smoking	3 mg MT from the 3rd to the 5th day until the trigger day	None	CPR, FR, No. of oocyte, MII
Nazzaro et al. 2011	Italy	Prospective	GnRH-ant IVF	112	PCOS; the 1 st IVF cycle, age 26–38	Previous pelvic surgery; endometriosis; hydrosalpins; uterine myomas; thrombophilic state	MI + FA + MT	MI + FA	CPR, No. of oocyte, MII, top quality embryo
Tunon et al. 2011	Spain	Double-blind randomized prospective	GnRH-ant/GnRH-a ICSI-ET Day 2–5 ET 1–3 embryos	120	Age 18-41ys; BMI 18–29; Normal ovulatory cycle 24–35 days	Azoospermia; abnormalities of the reproductive system; potential causes of ovulatory dysfunction; hypersensitivity to Gn	2 sachets containing 0.975 mg MT, 2 g MI, 200ug FA, and 27.5ug selenium for at least 2 months before the oocyte retrieval	NR	CPR, LBR, MR, No. of oocyte, MII, top quality embryo
Batioglu et al. 2012	Turkey	Prospective	GnRH-a IVF-ET ET time/number NR	85	Primary infertility; age 20–40; regular menstrual cycles 21-35days; no hormonal therapy for the last 3 months; no systemic illness	Serious endometriosis; serious male factor (azoospermia); hypogonatropic hypogonadism; bFSH > 13	3 mg MT Treatment time NR	None	CPR, FR, No. of oocyte, MII, top quality embryo
Nishihara et al. 2014	Japan	Self- control	GnRH-a IVF/ICSI-ET Day 2 ET	194 cycles in 97 patients	A poor oocyte and embryo quality in a previous IVF cycle (FR < 60%, had no good quality embryos, and had not reached to the blastocyst stage)	Age > 42; received MT at any time before enrollment	3 mg MT daily for at least 2 weeks, ending on the day of hCG injection	None	Maturation rate; FR;
Pacchiarotti et al. 2016	Italy	Double-blind RCT	GnRH-a ICSI-ET Day 2, ET number NR	331	bFSH < 12; Rotterdan criteria for PCOS; BMI 20–26; and the 1 st cycle	Declined to participate; tubal, uterine, genetics and male causes of infertility	MI(4 g), FA(400mcg) and MT(3 mg) from the 1 st day of the cycle until 14 days after ET	MI (4 g) and FA(400mcg)	CPR, MR, No. of oocyte, MII,
Jahromi et al. 2017 [8]	Iran	Double-blinded, RCT	GnRH-a protocol IVF-ET Day 3, ET number NR	66	The 1 st ART cycle, normal male factor and uterine cavity, and 2 of the criteria: (1)bilateral AFC ≤ 6; (2)AMH ≤ 1;(3) bFSH ≥ 10	Declined to participate, poor compliance, or poor responses to Gn	3 mg MT every night from the 5th day of one cycle prior to COS up to oocyte retrieval	Placebo	CPR, MR, No. of oocyte, MII, top quality
Fernando et al. 2018	Australia	Double-blind RCT	GnRH-ant IVF or ICSI-ET Day3 or 5, ET number NR	160	First cycle of IVF/ICSI; age 18–45; BMI 18–35; undergoing an GnRH-ant cycle	Untreated endometriosis, uterine malformations, large distorting fibroids or endometrial polyps, autoimmune disease, concurrent use of the other adjuvant therapies, malignancy, PGT, sensitivity to melatonin, or taking medications known to interact with MT	2/4/8 mg MT from day 2 until the day before oocyte retrieval	Placebo	LBR, CPR, MR, FR, No. of oocyte, top quality embryo
Espino et al. 2019 [7]	Spain		GnRH-ant ICSI-ET Day2 or 3 ET number 1	30	UI, normospermic, normal ovulation	< 18 years, active smokers, concurrently using other adjuvant therapies, autoimmune disorders/hypersensitivity to melatonin or its metabolites; unwilling to comply with the study procedures; misused alcohol in the preceding 3 months	3/6 mg MT from the first appointment to COS until oocyte retrieval	None	LBR, CPR, FR, MR, No. of oocyte, MII

*ET* Embryo transfer, *NR* No report,* MT *Melatonin, *MI* Myo-inositol,* FA* Folic acid, *FR* Fertilization rate, *CPR *Clinical pregnancy rate, *BPR* Biochemical pregnancy rate,* LBR* Live birth rate

Among the 11 included studies, 1 study involved infertile women with sleep disturbance (60 participants), 2 studies focused on women with polycystic ovary syndrome (PCOS; 443 participants), 3 studies on women with normal ovarian function (216 participants), 1 study on women with decreased ovarian reserve (DOR; 66 participants), and 3 studies on women with a history of low fertilization rate or poor-quality embryo (277 participants). One study did not report participant characteristics.

### Meta-analysis

A total of 10, 3, 6, 7, 9, 8 and 6 studies were included in the meta-analysis to assess the effect of MT supplementation on CPR, LBR, MR, FR, number of oocytes retrieved, number of MII oocytes, and number of top-quality embryos, respectively.

**Fig. 1:**
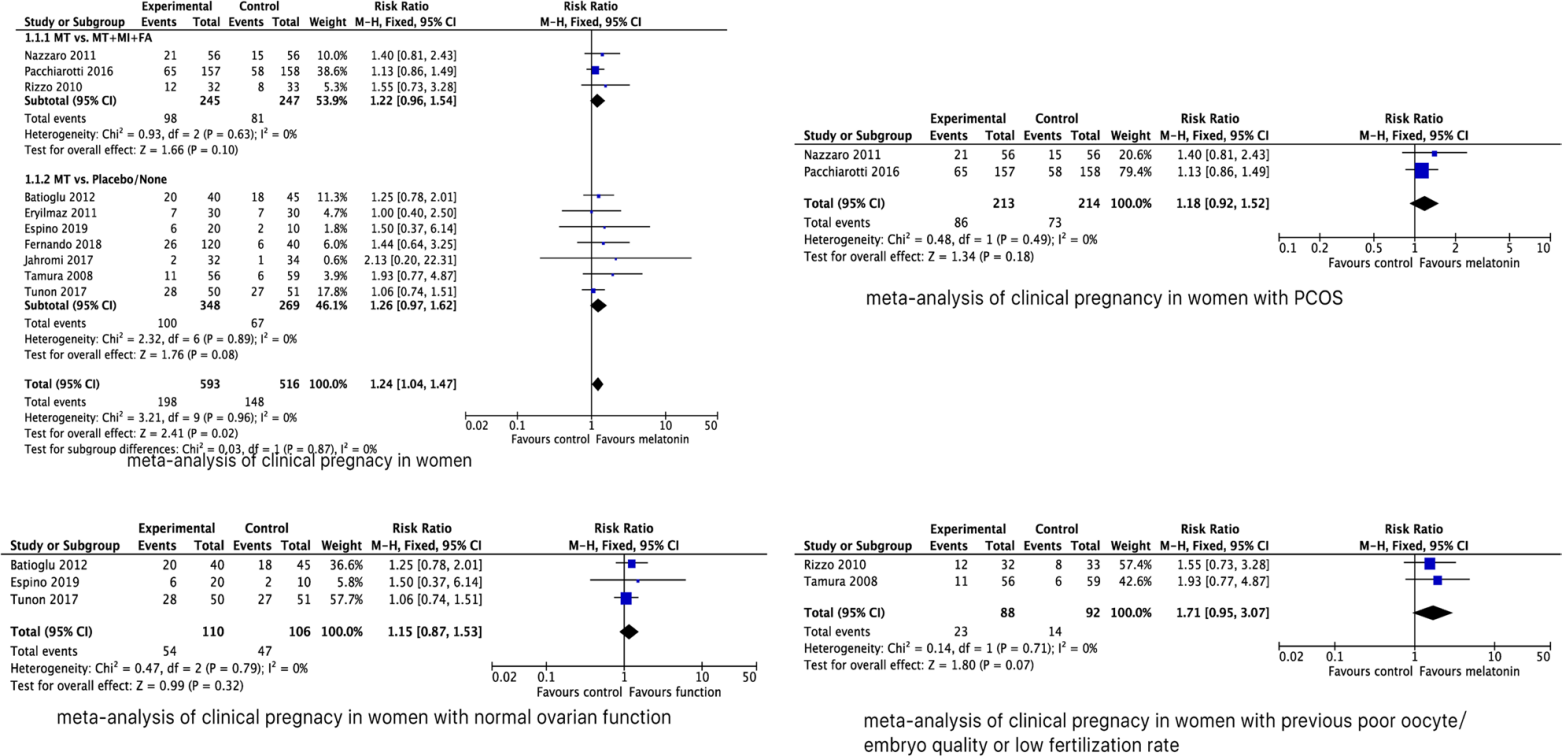
Forest plot showing the results of meta-analysis of studies comparing the effect of MT supplementation on clinical pregnancy rate following IVF/ICSI

To evaluate the effect of MT supplementation on CPR, 10 studies were analyzed, including 593 ART cycles in the MT supplementation group and 516 cycles in the control group. The CPR was significantly increased in women with MT supplementation compared to control group. No heterogeneity was observed across studies as *P* = 0.96 (l^2^ = 0%). The fixed effects model was implied and the combined PR was 1.24 (95% CI, 1.04, 1.47; *P* = 0.02) (Fig. 1).

Subsequently, we presented stratified results based on the interventions. The MT supplementation, whether or not combined with MI + FA, did not bring beneficial effect on CPR with combined RR 1.22 (95% CI, 0.96, 1.54; *P* = 0.10) compared to MI + FA alone, and 1.26 (95% CI, 0.97, 1.62; *P* = 0.08) compared to placebo or no treatment. Furthermore, the included studies were stratified into 4 subgroups based on participant characteristics: women with PCOS, normal ovarian function, a history of poor oocyte quality or low fertilization rate, and DOR. The pooled RRs in women with PCOS, normal ovarian function, and a history of poor oocyte quality or low fertilization rate were 1.18 (95% CI, 0.92, 1.52; *P* = 0.18), 1.15 (95% CI, 0.87, 1.53; *P* = 0.32) and 1.71 (95% CI, 0.95, 3.07; *P* = 0.07), respectively, with no significant difference compared to the control group.

**Fig. 2:**
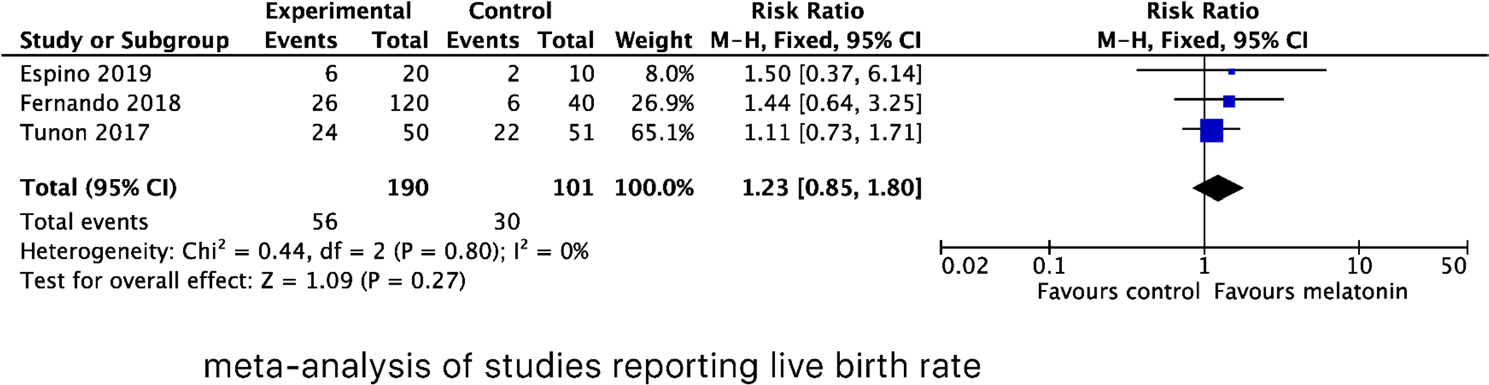
Forest plot showing the results of meta-analysis of studies comparing the effect of MT supplementation on live birth rate following IVF/ICSI

Three studies were included to evaluate the effects of MT supplementation on the LBR, including 190 cycles in the MT supplementation group and 101 cycles in the control group. The LBR was comparable between the intervention and the control group. The statistical heterogeneity was low with *P* = 0.80 (l^2^ = 0%). The combined RR was 1.23 (95% CI, 0.85, 1.80; *P* = 0.27) (Fig. 2).

**Fig. 3:**
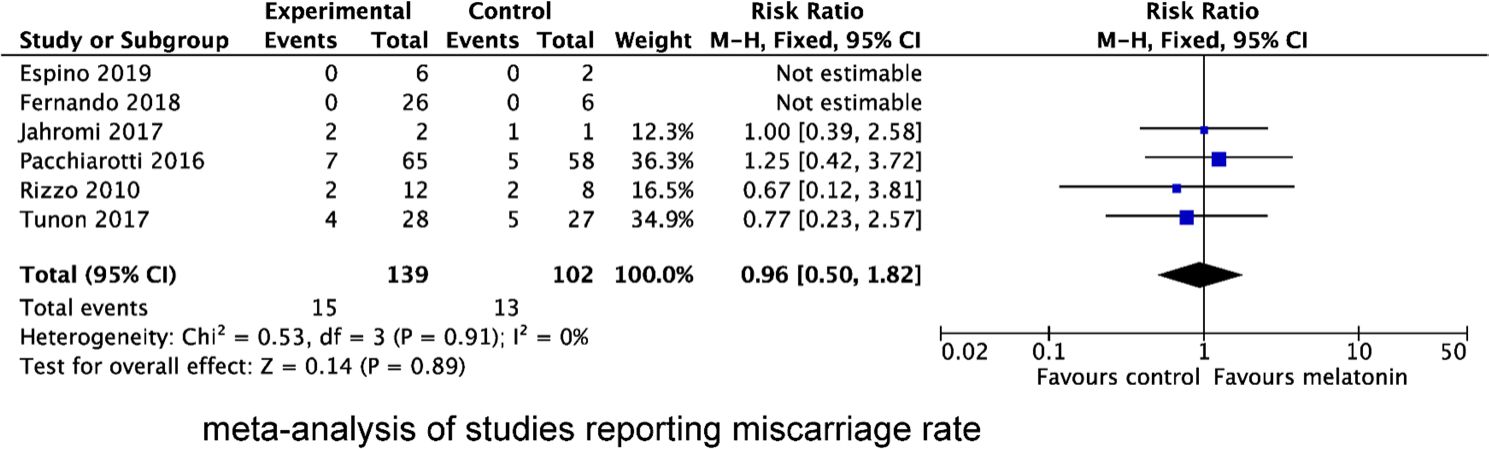
Forest plot showing the results of meta-analysis of studies comparing the effect of MT supplementation on miscarriage rate following IVF/ICSI

Of the 11 included studies, six evaluated the effect of MT supplementation on MR, involving 139 participants in the MT supplementation group and 102 in the control group. The pooled results failed to show any significant benefit in reducing MR. The pooled RR for MR was 0.96 (95% CI, 0.50, 1.82; *P* = 0.89) with fixed effects model. No heterogeneity was observed among these studies (l^2^ = 0%, *P* = 0.91. Figure 3).

**Fig. 4:**
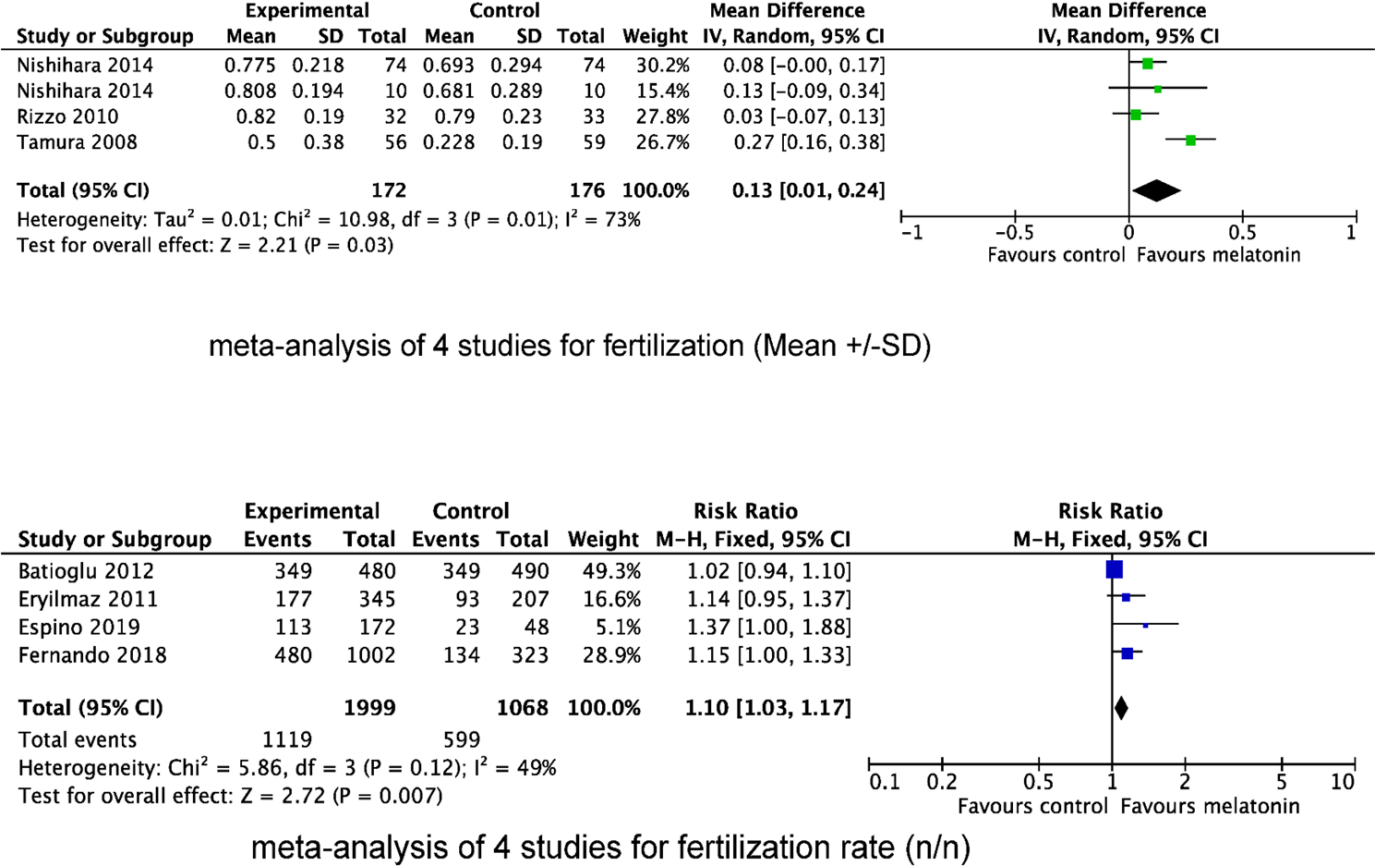
Forest plot showing the results of meta-analysis of studies comparing the effect of MT supplementation on fertilization rate following IVF/ICSI

Three studies reported FR as mean ± SD, yielding a pooled MD of 0.13 (95% CI, 0.01, 0.24; *P* = 0.03), with moderate statistical heterogeneity (l^2^ = 73%, *P* = 0.01). In addition, four studies assessed FR as cases (events/total), resulting in a pooled RR of 1.10 (95% CI, 1.03, 1.17; *P* = 0.007) with moderate statistical heterogeneity (l^2^ = 49%, *P* = 0.12). (Fig. 4)

**Fig. 5:**
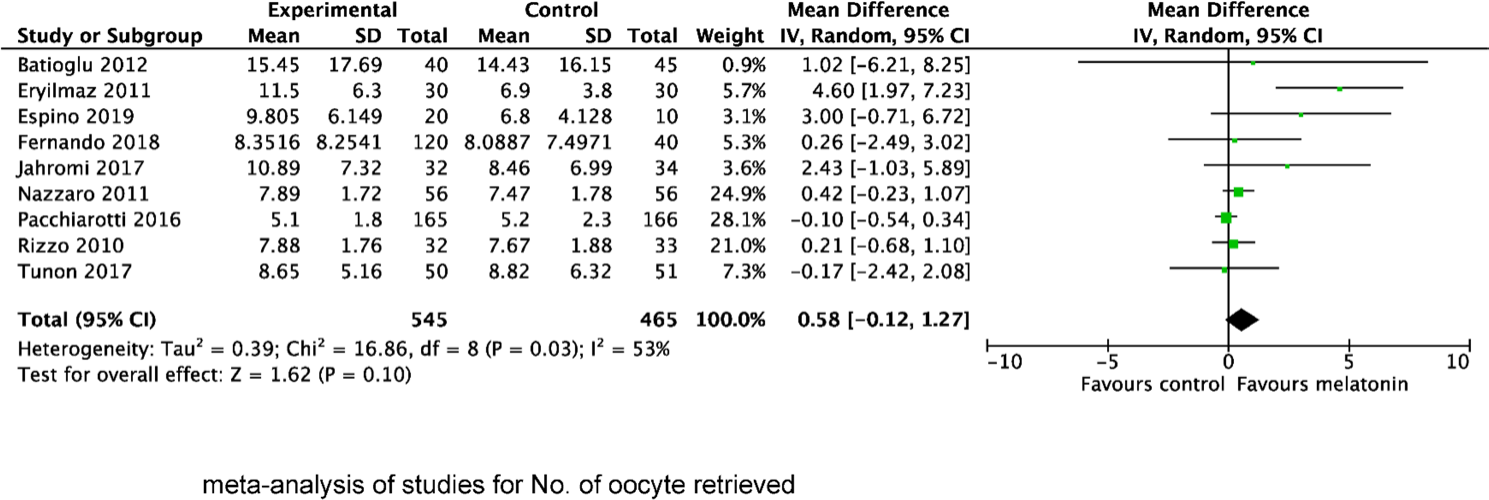
Forest plot showing the results of meta-analysis of studies comparing the effect of MT supplementation on the number of oocytes retrieved following IVF/ICSI

**Fig. 6:**
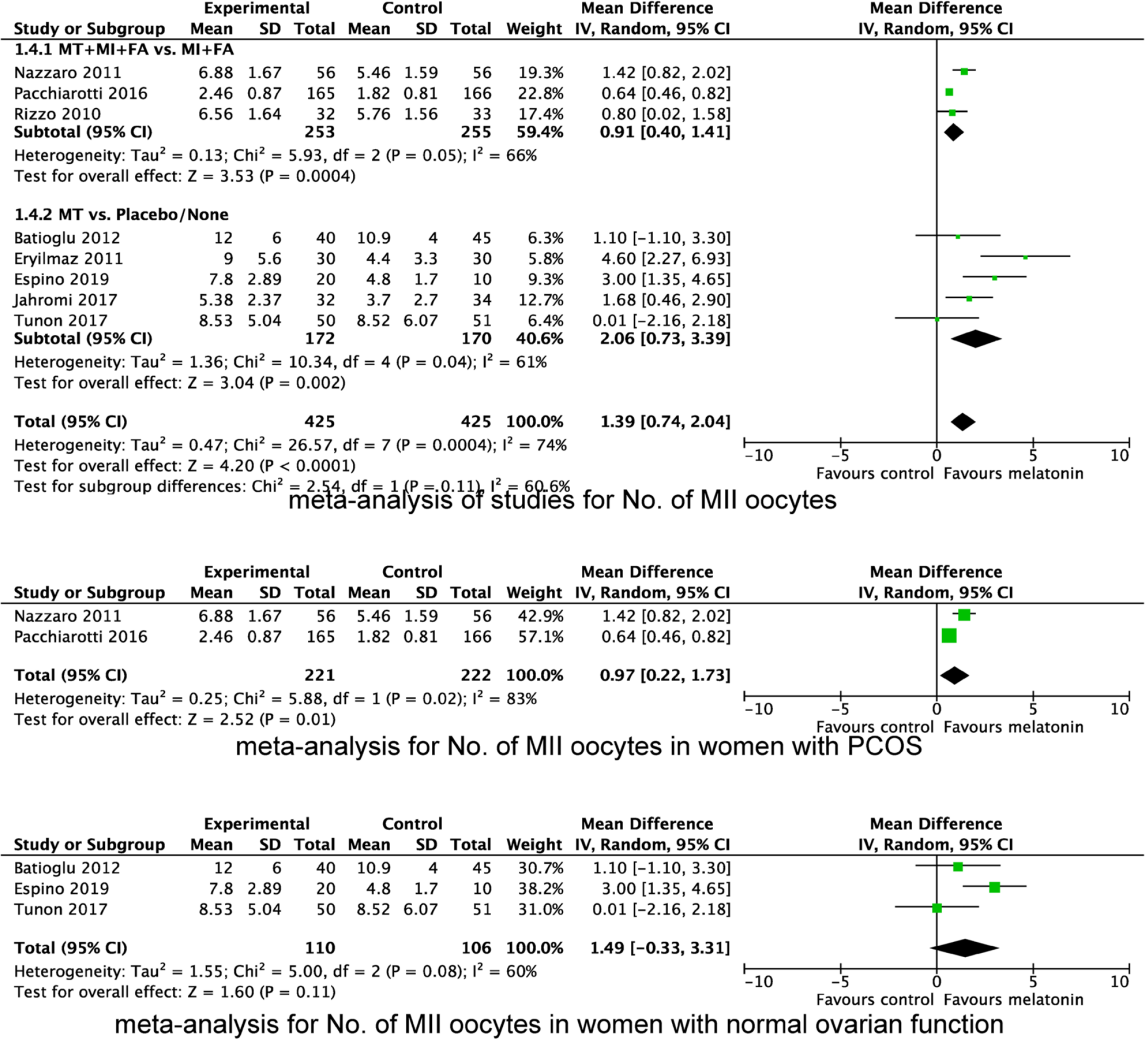
Forest plot showing the results of meta-analysis of studies comparing the effect of MT supplementation on the number of MII oocytes following IVF/ICSI

**Fig. 7:**
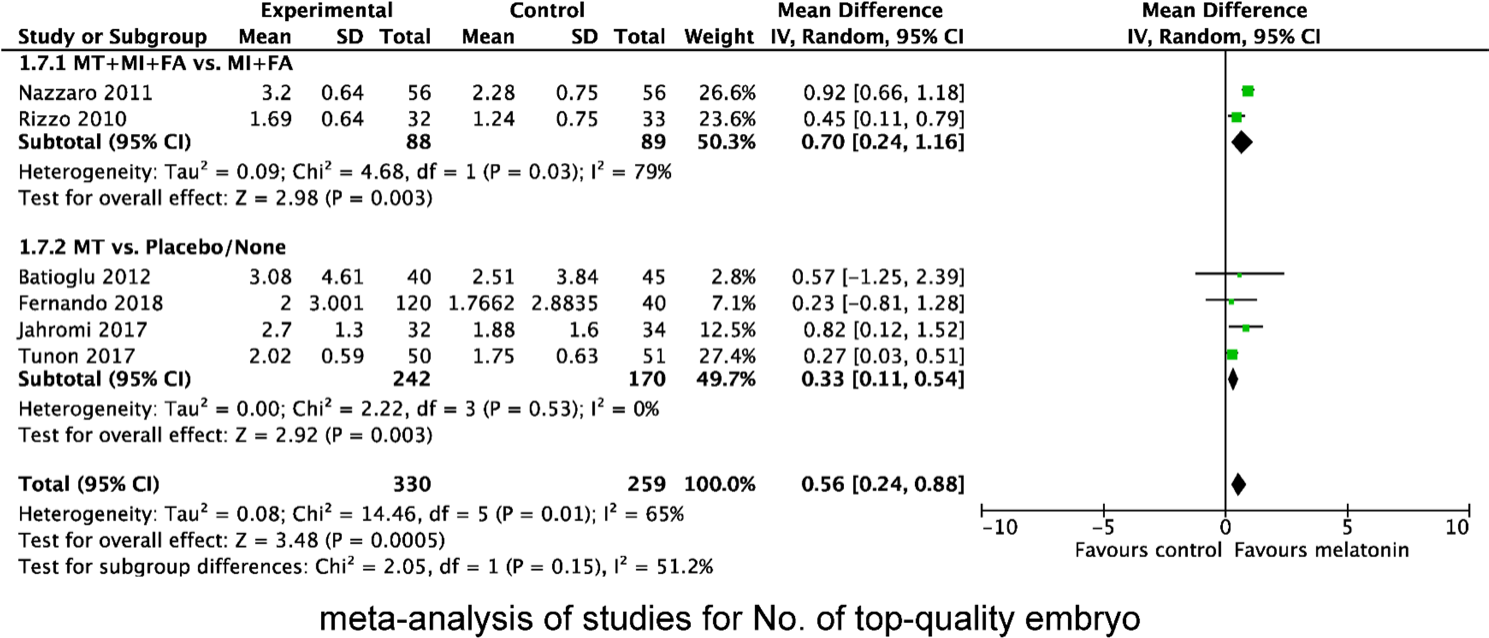
Forest plot showing the results of meta-analysis of studies comparing the effect of MT supplementation on the number of top-quality embryos following IVF/ICSI

We also evaluated the effect of MT supplementation on the number of retrieved oocytes (Fig. 5), MII oocytes (Fig. 6) and top-quality embryos (Fig. 7). The result showed that MT supplementation increased number of MII oocytes (MD 1.39; 95% CI, 0.74, 2.04; *P* < 0.0001) and top-quality embryos (MD 0.56; 95% CI, 0.24, 0.88; *P* = 0.0005) compared to control group. However, there was no difference in the number of oocytes retrieved (MD 0.58; 95% CI, −0.12, 1.27; *P* = 0.10). Regardless of whether MT was administered with or without MI + FA, MT supplementation significantly increased the number of MII oocytes (MI + MT + FA vs. MI + FA: MD 0.91; 95% CI 0.40, 1.41; *P* = 0.004; MT vs. placebo/no treatment: MD 2.06, 95% CI 0.73, 3.39; *P* = 0.002), and top-quality embryos (MI + MT + FA vs. MI + FA: MD 0.70; 95% CI 0.24, 1.16; *P* = 0.003; MT vs. placebo/no treatment: MD 0.33, 95% CI 0.11, 0.54; *P* = 0.003). However, subgroup analysis revealed that the increase in the number of MII oocytes was significant only in women with PCOS (MD 0.97; 95% CI 0.22, 1.73; *P* = 0.0004), but not in those with normal ovarian function (MD 1.49; 95% CI −0.33, 3.31; *P* = 0.11), when compared with the control group.

All studies included were rated as having medium to high quality based on the Newcastle-Ottawa Quality Assessment Scale (data not shown). No publication bias was observed in the funnel plot evaluating the effect of MT supplementation on CPR, LBR, MR, number of oocytes retrieved, MII oocytes, and top-quality embryos, as indicated by their symmetrical distribution. However, funnel plot asymmetry suggested a modest publication bias when assessing the effect of MT supplementation on FR. (Sup Fig. 1–7)

## Discussion

To date, two systematic reviews have evaluated the impact of MT supplementation on the outcomes of IVF/ICSI treatments, but have reported conflicting conclusions. To the best of our knowledge, this study included the most comprehensive dataset with the largest sample size of 1303 IVF/ICSI cycles.

In the present systematic review, 10, 3, 6, 7, 9, 8, and 6 studies were included to assess the effect of MT supplementation on the CPR, LBR, MR, FR, number of oocytes retrieved, MII oocytes, and top-quality embryos following IVF/ICSI, respectively. Ther results indicated that MT supplementation was associated with significant improvements in CPR (combined RR 1.24; 95% CI 1.04, 1.47; *P* = 0.02), FR (3 studies: MD 0.13; 95% CI, 0.01, 0.24; *P* = 0.03; 4 studies: RR 1.10; 95% CI, 1.03, 1.17; *P* = 0.007), number of MII oocytes(MD 1.39; 95% CI, 0.74, 2.04; *P* < 0.0001), and top-quality embryos (MD 0.56; 95% CI, 0.24, 0.88; *P* = 0.0005). However, no significant differences were found in LBR, MR or the number of oocytes retrieved.

Nonetheless, when the studies were stratifies by the interventions or by participant characteristics, the results differed notably: (1) No significant differences in CPR were observed, regardless of the interventions used or participant subgroup; (2) MT supplementation significantly increased the number of MII oocytes and top-quality embryos regardless of the interventions; (3) a significant increase in the number of MII oocytes was observed in women with PCOS, but not in those with normal ovarian function.

The conclusions of the present study were not entirely consistent with the systematic reviews by Seko et al. [10] and Hu et al. [11]. The former, which included only 5 studies and did not report LBR, found no beneficial effect of MT supplementation on CPR or the number of oocytes retrieved. The latter, which incorporated one intra-uterine insemination (IUI) study [3] and one study involving in-vitro application of MT [12], suggested that MT supplementation increased CPR, number of oocytes, MII oocytes, and good-quality embryos, but did not improve LBR in ART cycles. However, this study included incomplete data, and employed an inappropriate method for the calculation of miscarriage rate. The dosage of MT varied considerably across the included studies, ranging from 2 mg to 8 mg daily, with two trials specifically explored dose-dependent effects [7, 13]. However, no consistent dose-response relationship was observed.

During the ART process, controlled ovarian stimulation (COS), in vitro incubation of oocyte and embryo, and fertilization may expose oocytes and embryos to a elevated level of ROS compared to the physiological condition. Excessive ROS can induce telomere damage [14], compromise cell membrane integrity, and change functional structures (14) ultimately resulting in impaired oocyte/embryo quality and suboptimal pregnancy outcome. Therefore, mitigating the oxidative stress induced by ROS is essential to the development of high-quality embryos.

Antioxidant treatment is one of the key strategies to counteract the oxidative stress. The effects of MT on female reproduction has been comprehensively reviewed by Tamura et al. [15]. Both animal and human studies have indicated that MT may be a promising agent in the treatment of infertility [13, 16]. Several clinical trials have reported that MT supplementation improves the quality of oocyte and embryo, potentially enhancing IVF outcomes [6–8]. However, other studies have failed to show such beneficial effects, leading to conflicting conclusions [9, 17].

Consistent with previous studies, our pooled results indicate that MT supplementation has beneficial effects on oocyte and embryo quality, particularly in women with PCOS. As is well established, one of the hallmark futures of PCOS is oocyte maturation disorder [18]. Several potential mechanisms may explain these findings: (1) MT receptors (MTR) have been identified in granulosa cells, oocytes and embryos [19–21], providing a biological foundation for MT’s effects; (2) MT is amphiphilic, enabling it to readily pass through cell membranes [4]. (3) Both MT and its metabolites are terminal antioxidant that do not undergo redox cycling and therefore do not act as pro-oxidant under any conditions [22, 23]. (4) MT’s known psychological effects, such as improved sleep and reduced anxiety, may indirectly benefit IVF outcomes [17].

In addition to its powerful free-radical scavenging effect, MT exhibits immunomodulatory effect, promotes progesterone secretion, and inhibits the synthesis of prostaglandins-a process that may otherwise increase the risk of miscarriage and premature delivery [24]. An animal study on rats has shown that MT can enhance the expression of MT receptor and p53 receptor, thereby influencing endometrial morphology and promoting embryo implantation [25]. Theoretically, MT may contribute to both the establishment and maintenance of pregnancy. In cases of spontaneous abortion not attributed to chromosomal or uterine abnormalities, MT has been reported as a potential protective factor [4]. Regrettably, our results did not demonstrate a statistically significant beneficial effect of MT supplementation on CPR, MR, and LBR.

We further analyzed several possible explanations: (1) MT has a rapid metabolism and short half-life, which may result in suboptimal serum concentration from a single daily dose during IVF cycles, thereby limiting its antioxidant efficacy. (2) The timing of initiation and duration of MT supplementation varied across studies, potentially influencing treatment outcomes. (3) The success of pregnancy following IVF/ICSI is affected by multiple factors, and potential benefits of MT may have been counterbalanced by other confounding or adverse influences.

A key strength of systematic reviews is the comprehensive evaluation of the findings of individual studies. The present systematic review indicated that MT supplementation exerts a positive effect on FR, as well as on quality of oocyte and embryo following IVF/ICSI. Our study included the largest sample size to date and was the first to specially evaluate fertilization outcomes. Additionally, we made every effort to obtain complete data by contacting with authors and calculating missing values from published information.

Admittedly, this meta-analysis has certain limitations, the most notable being the variability among the included studies. Key variations included differences in participant characteristics, interventions (MI + FA + MT, MT), MT dose and treatment duration, inclusion/exclusion criteria, and outcome measures. The observed heterogeneity across outcomes likely reflects underlying differences in these potential effect modifiers. Moreover, some potential confounding factors, such as smoking status and the number of previous failed IVF cycle, were not adequately controlled. Although all included studies were randomized controlled trials or well-designed prospective studies, variations in participant characteristics and study methodologies may still have introduced residual confounding, potentially affecting the pooled outcomes. While definitive subgroup effects remain unclear, the variation highlights the need to identify which patient populations may benefit most from MT supplementation and under what dosing conditions. This review reflects studies available at the time of the original search, and findings should be interpreted with the possibility that newer evidence may change the conclusions. Future updates of this meta-analysis will be essential as additional high-quality evidence becomes available.

## Conclusion

This systematic review suggests that MT supplementation may have beneficial effects on oocyte maturation, fertilization, and embryo quality. However, current evidence does not demonstrate a significant effect of MT supplementation on LBR, CPR, or MR. Based on these finds, MT supplementation may be considered, particularly for women with PCOS or a history of poor oocyte quality or low fertilization rates.

## Supplementary Information

Supplementary Material 1.

Supplementary Material 2.

Supplementary Material 3.

Supplementary Material 4.

Supplementary Material 5.

Supplementary Material 6.

Supplementary Material 7.

Supplementary Material 8.

Supplementary Material 9.

## Data Availability

All data generated or analyzed during the current study are included in this published article. This review was not registered in a systematic review database.

## Abbreviations

ART: Assisted reproductive technology
IVF-ET: In vitro fertilization - embryo transfer
ICSI: Intracytoplasmic sperm injection
MT: Melatonin
RR: Risk Ratio
MD: Mean difference
CPR: Clinical pregnancy rate
LBR: Live birth rate
MR: Miscarriage rate
FR: Fertilization rate
CI: Confidence interval
MI: Myoinositol
FA: Folic acid
DOR: Decreased ovarian reserve
PCOS: Polycystic ovary syndrome
ROS: Reactive oxygen species
