# Supplementary figures and images for: Melatonin supplementation and outcomes of assisted reproductive technology: a systematic review and meta-analysis
Source: BMC Pregnancy Childbirth. 2025 Nov 25;26:9. doi: 10.1186/s12884-025-08503-1 (PMC12764091; doi:10.1186/s12884-025-08503-1)

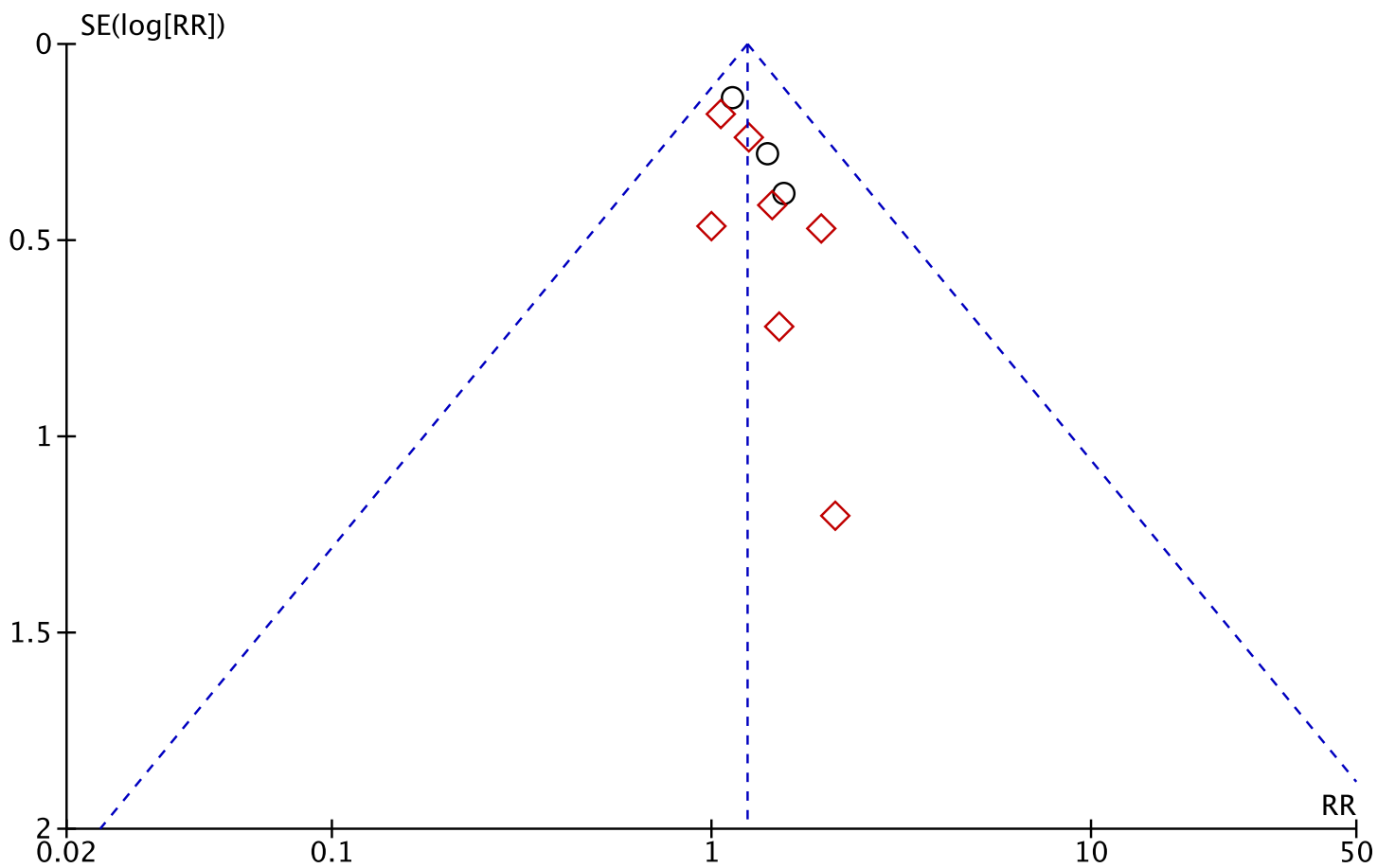

**Subgroups**

○ MT vs. MT+MI+FA

◇ MT vs. Placebo/None

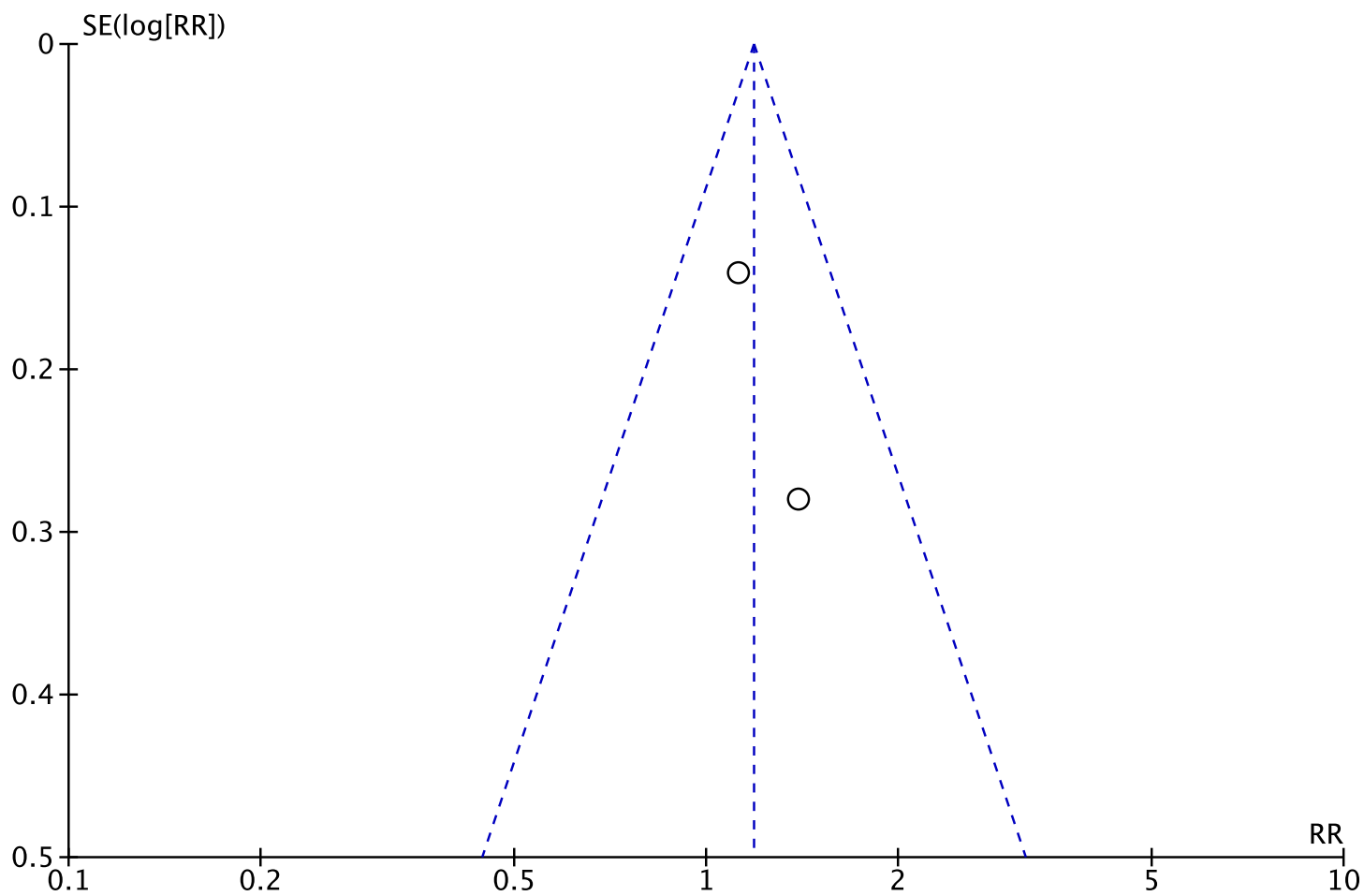

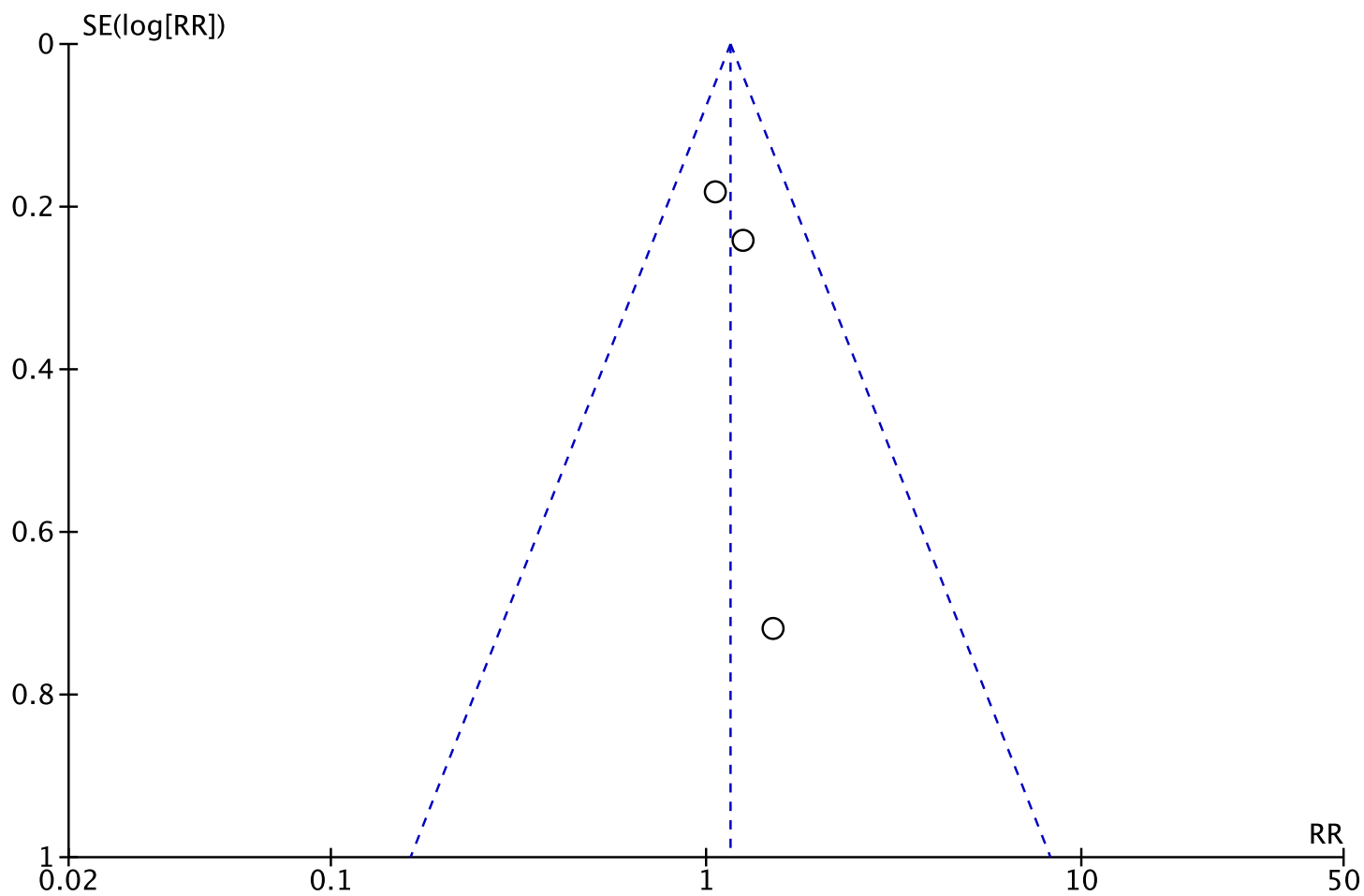

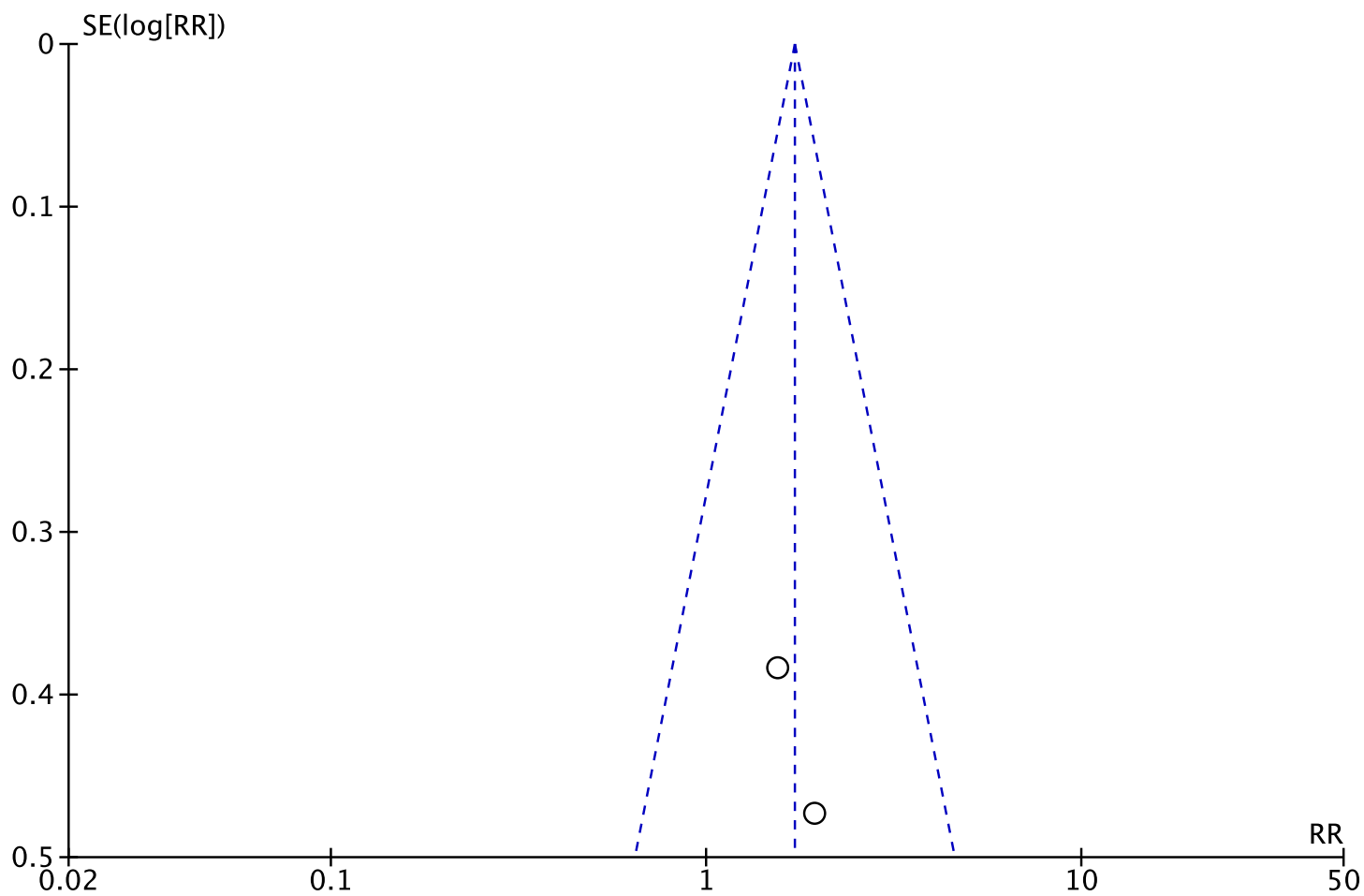

Supplement: Supplementary file 1 — Supplementary Material 1. [file 12884_2025_8503_MOESM1_ESM.pdf]

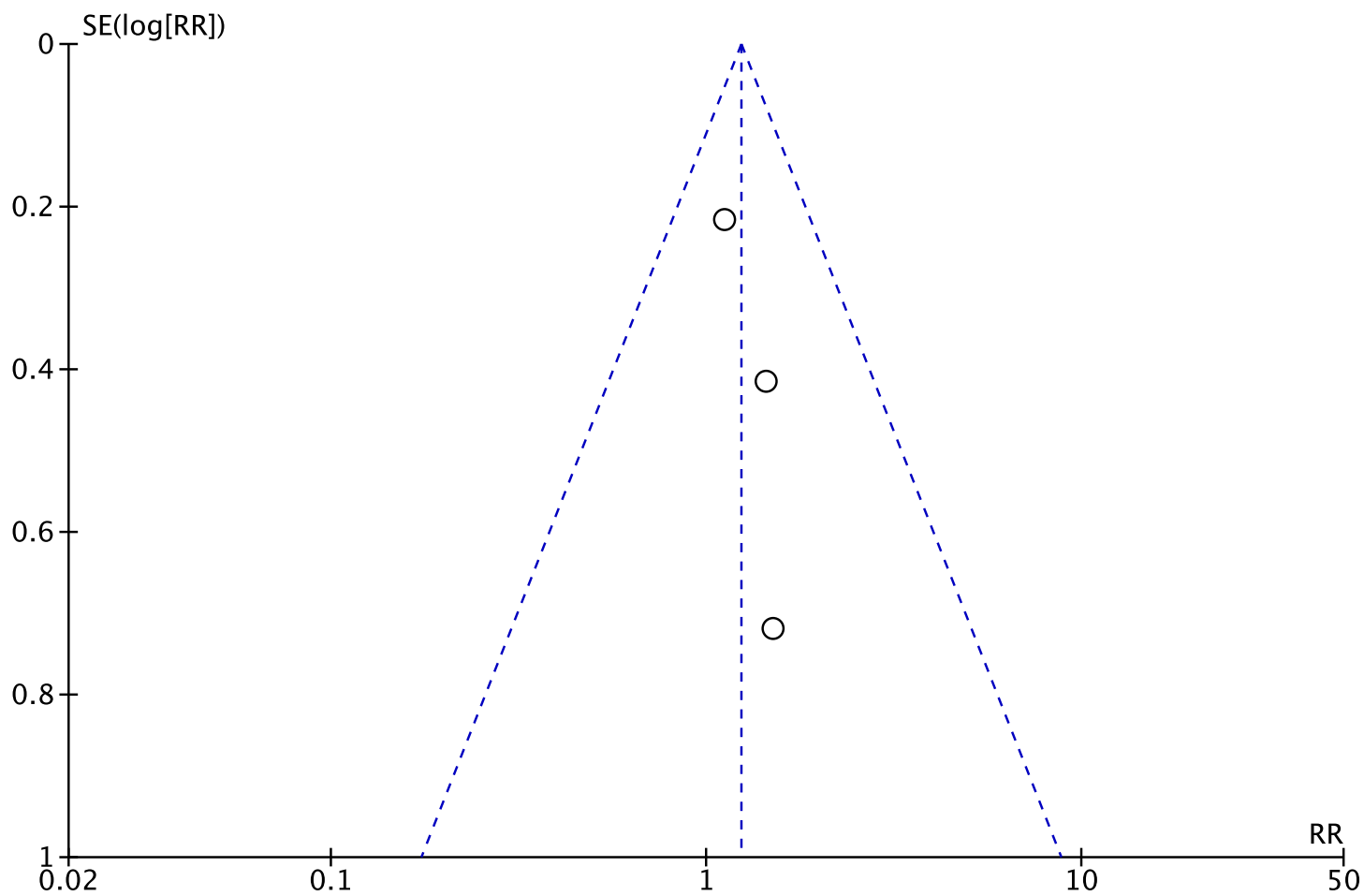

Supplement: Supplementary file 2 — Supplementary Material 2. [file 12884_2025_8503_MOESM2_ESM.pdf]

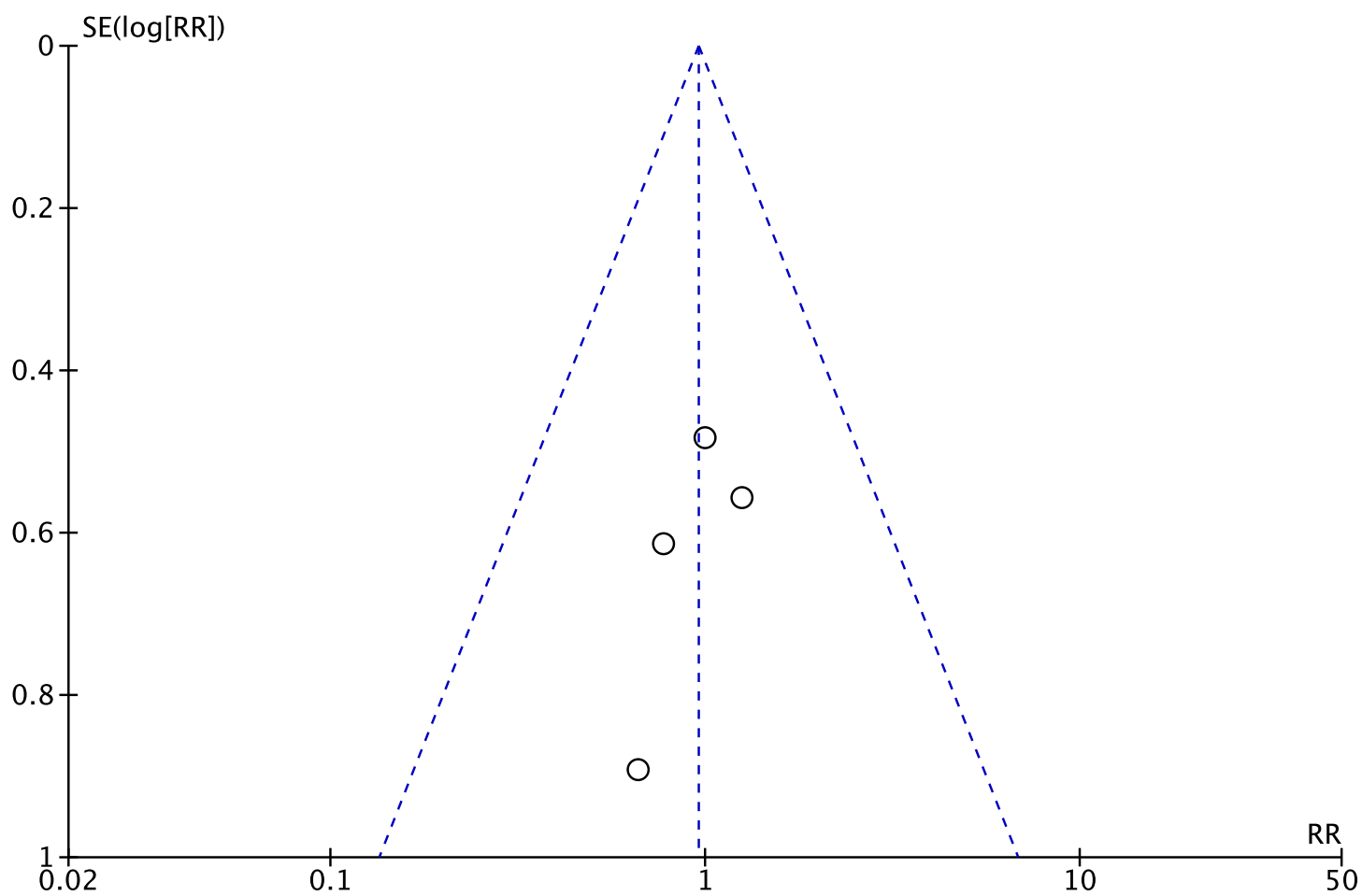

Supplement: Supplementary file 3 — Supplementary Material 3. [file 12884_2025_8503_MOESM3_ESM.pdf]

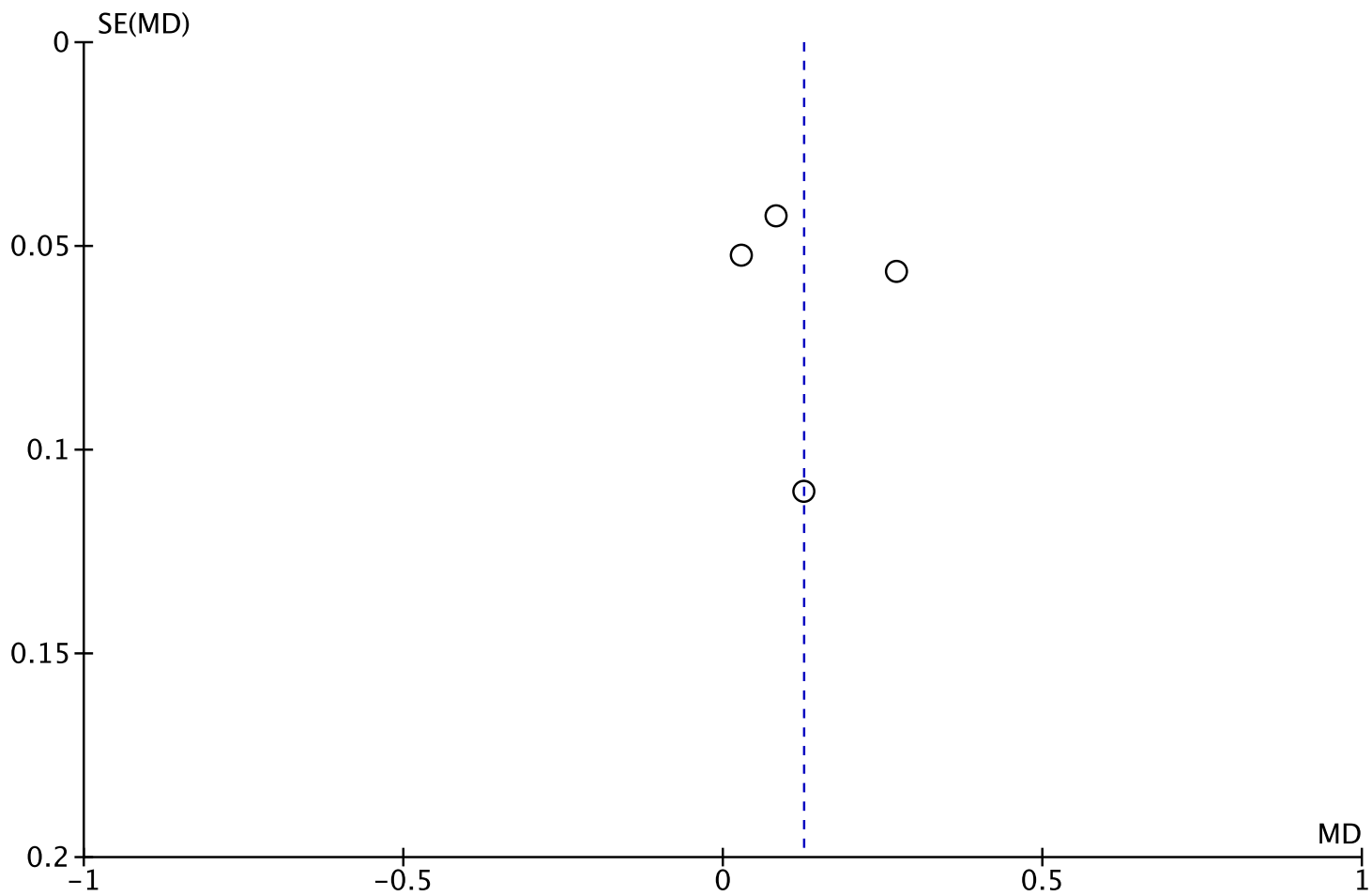

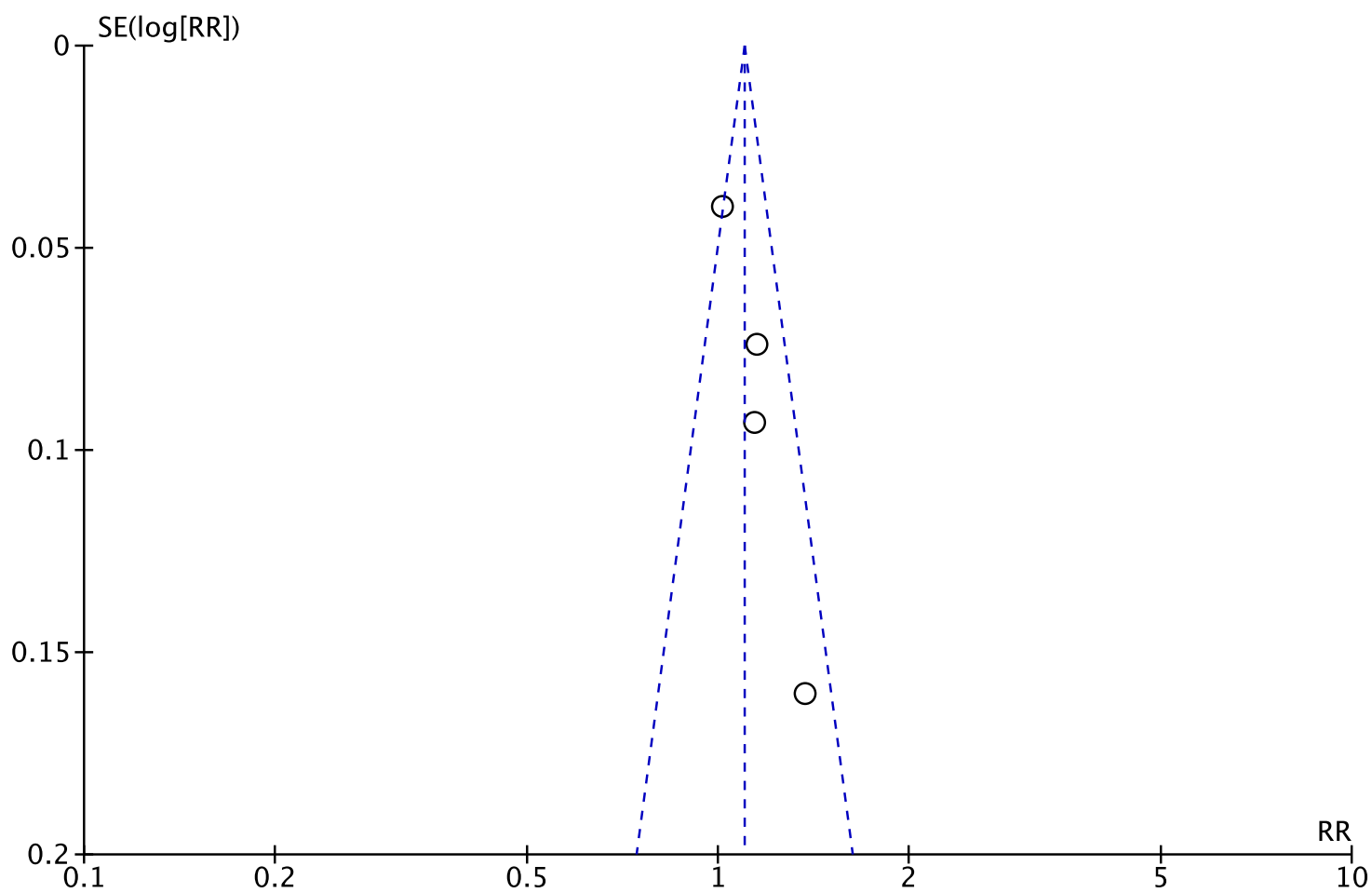

Supplement: Supplementary file 4 — Supplementary Material 4. [file 12884_2025_8503_MOESM4_ESM.pdf]

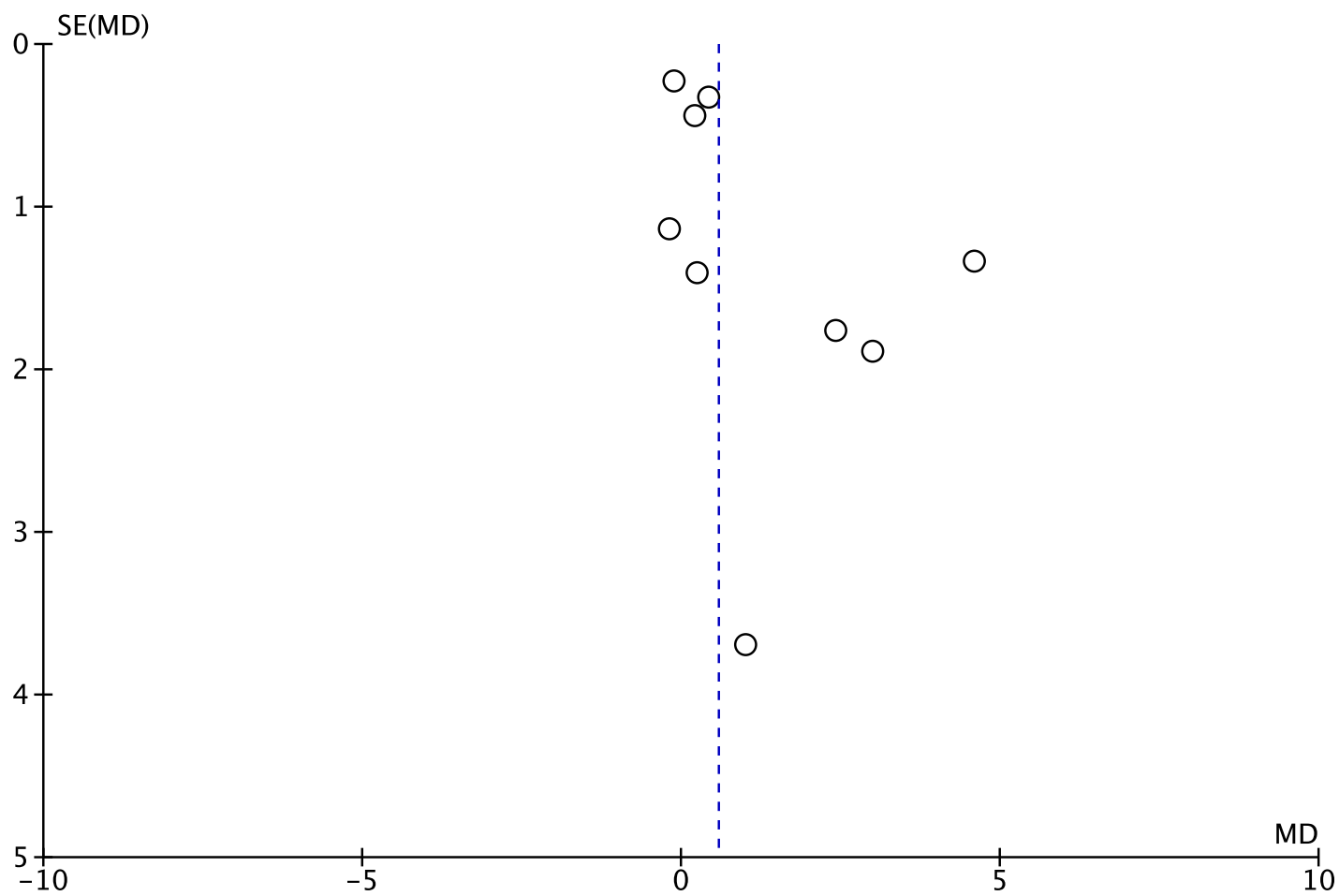

Supplement: Supplementary file 5 — Supplementary Material 5. [file 12884_2025_8503_MOESM5_ESM.pdf]

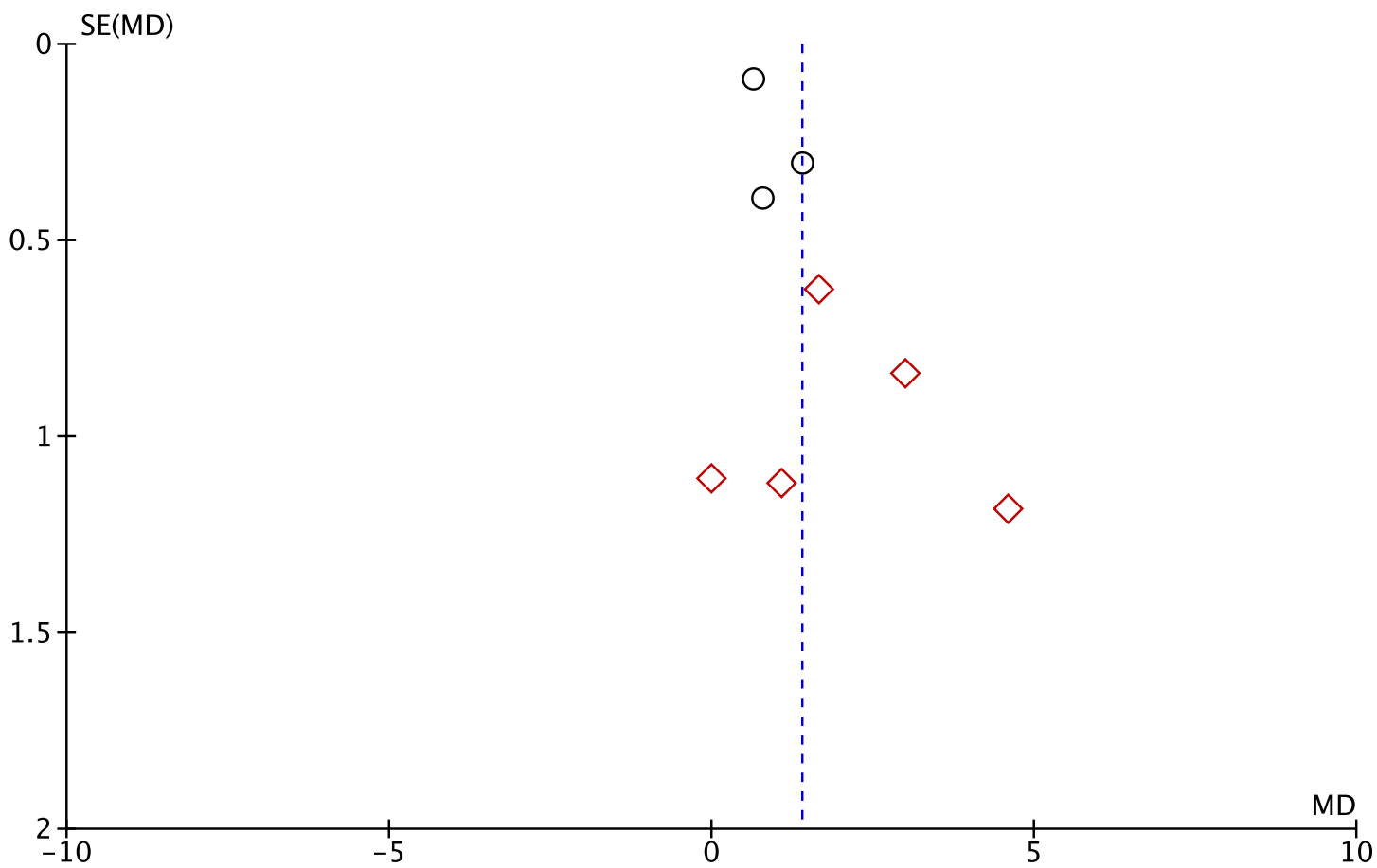

**Subgroups**

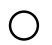

MT+MI+FA vs. MI+FA

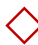

MT vs. Placebo/None

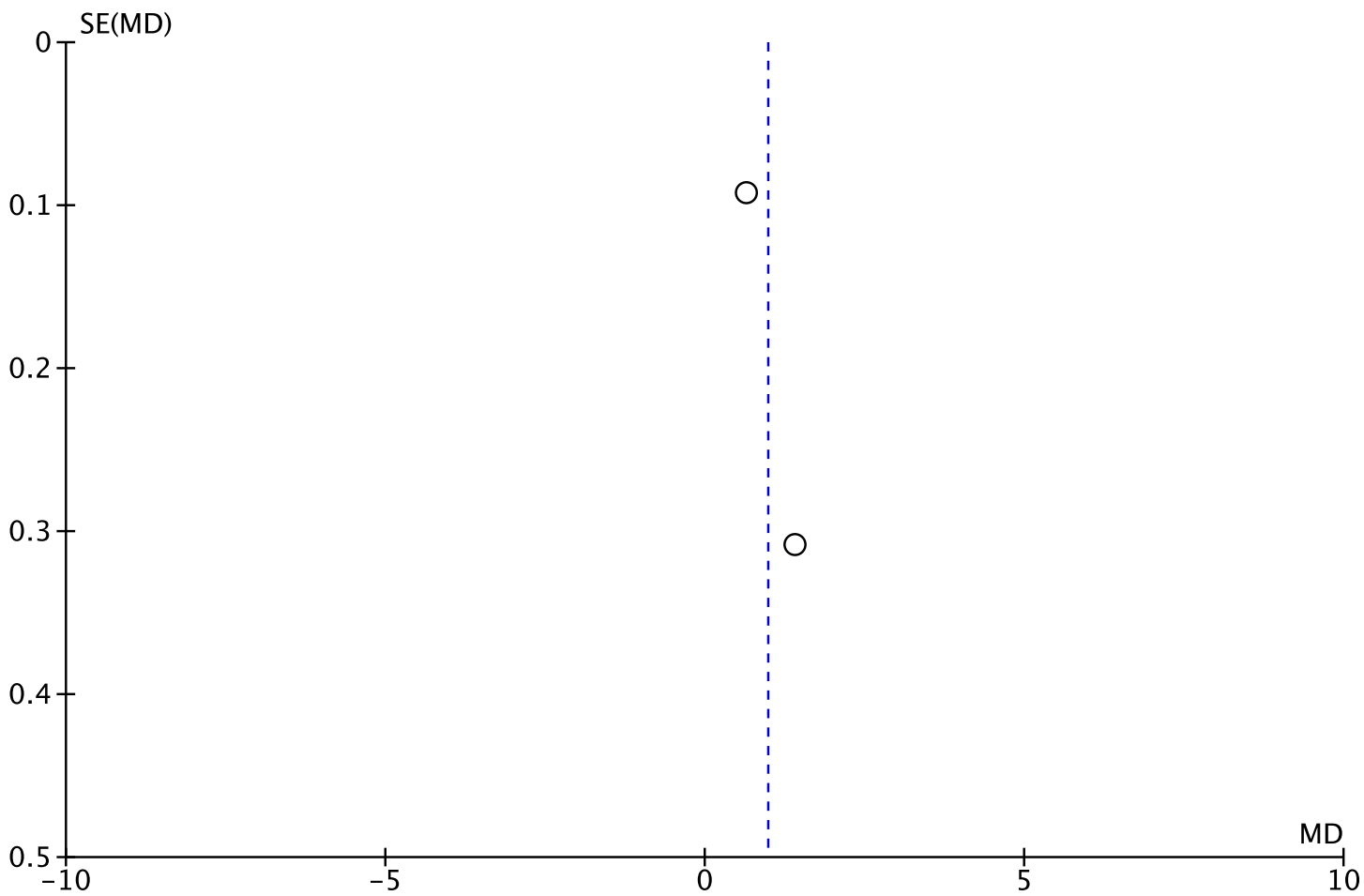

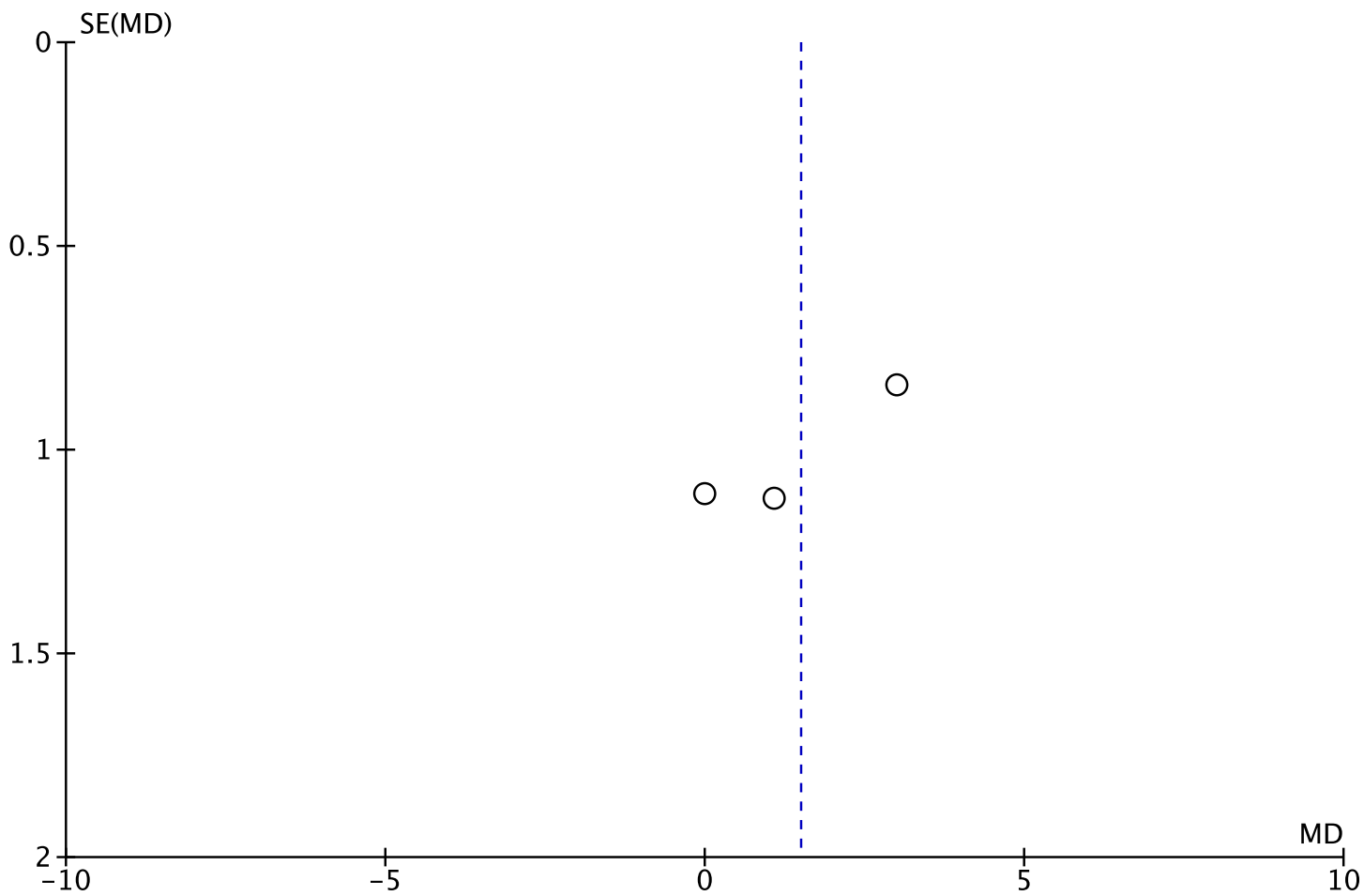

Supplement: Supplementary file 6 — Supplementary Material 6. [file 12884_2025_8503_MOESM6_ESM.pdf]

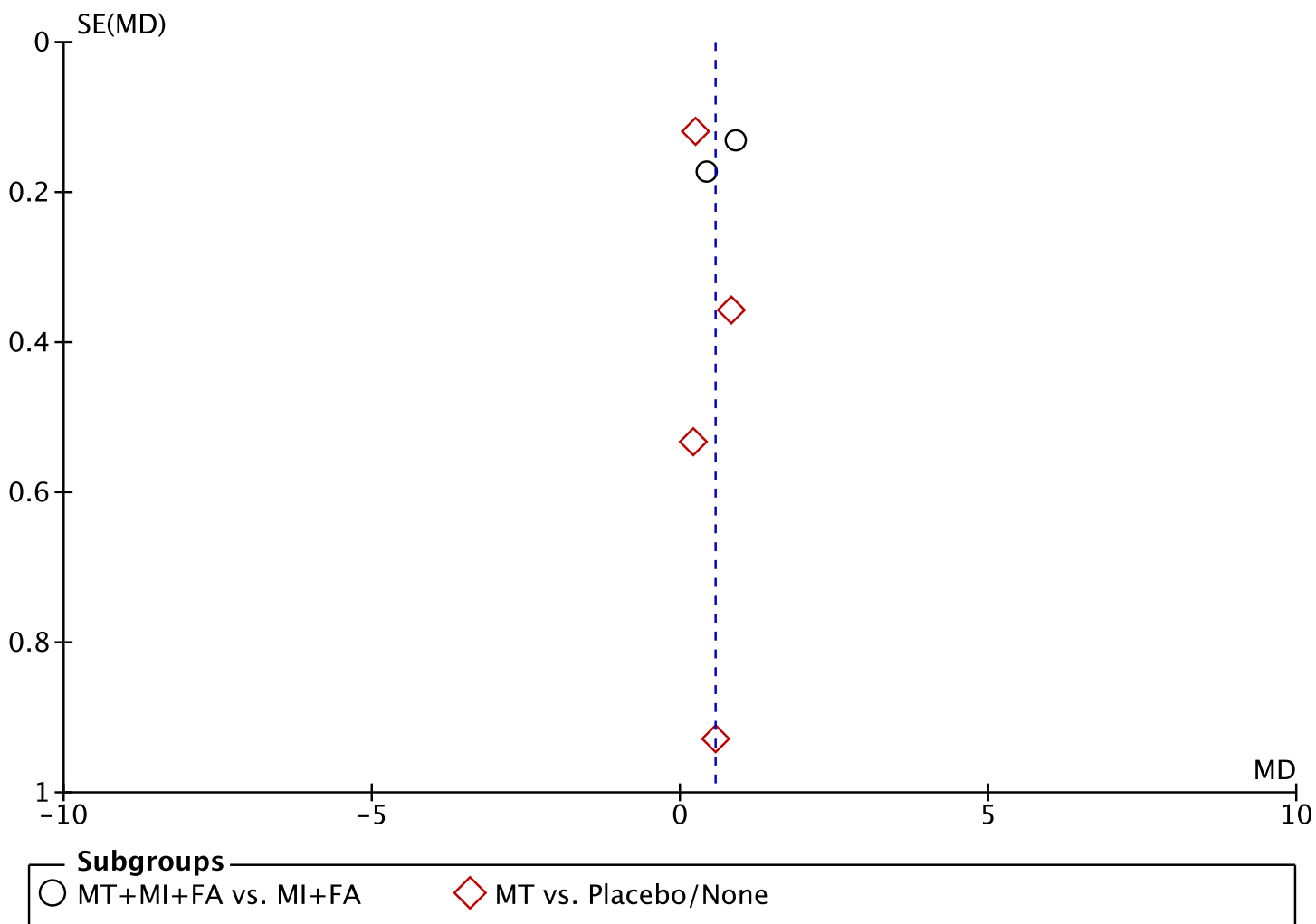

Supplement: Supplementary file 7 — Supplementary Material 7. [file 12884_2025_8503_MOESM7_ESM.pdf]
