# Supplementary material for: Melatonin supplementation and outcomes of assisted reproductive technology: a systematic review and meta-analysis
Source: BMC Pregnancy Childbirth. 2025 Nov 25;26:9. doi: 10.1186/s12884-025-08503-1 (PMC12764091; doi:10.1186/s12884-025-08503-1)
Supplement: Supplementary file 8 — Supplementary Material 8. [file 12884_2025_8503_MOESM8_ESM.docx]

**Supplementary information**

**Supplemental Figure 1** Funnel plot of analysis for the effect of MT supplementation on clinical pregnancy rate

**Supplemental Figure 2** Funnel plot of analysis for the effect of MT supplementation on live birth rate

**Supplemental Figure 3** Funnel plot of analysis for the effect of MT supplementation on miscarriage rate

**Supplemental Figure 4** Funnel plot of analysis for the effect of MT supplementation on fertilization rate

**Supplemental Figure 5** Funnel plot of analysis for the effect of MT supplementation on No. of oocyte

**Supplemental Figure 6** Funnel plot of analysis for the effect of MT supplementation on No. of MII

**Supplemental Figure 7** Funnel plot of analysis for the effect of MT supplementation on No. of embryo
