# Supplementary material for: Melatonin supplementation and outcomes of assisted reproductive technology: a systematic review and meta-analysis
Source: BMC Pregnancy Childbirth. 2025 Nov 25;26:9. doi: 10.1186/s12884-025-08503-1 (PMC12764091; doi:10.1186/s12884-025-08503-1)
Supplement: Supplementary file 9 — Supplementary Material 9. [file 12884_2025_8503_MOESM9_ESM.doc]

Supplemental Table 1 Flow chart showing study selection process

**Screening**

**Included**

**Eligibility**

**Identification**

|  |  |
| --- | --- |

Records identified through database searching
(n =160 )

Additional records identified through other sources
(n = 0 )

Records after duplicates removed
(n = 160)

Records screened
(n = 57 )

Records excluded
(n =103)

Full-text articles assessed for eligibility
(n =11)

Reasons of exclusion:
case report n=5

Review n=6

Without control group n=13

Cell/Animal study n=22

Studies included for clinical pregnancy
(n = 10)

Studies included for fertilization
(n = 7)

Studies included for live birth
(n =3)

Studies included for miscarriage
(n = 6)

Studies included for Number of Oocyte
(n = 9)

Studies included for Number of MII
(n = 8)

Studies included for Number of Top embryo
(n = 6)
